# Improving children’s fundamental movement skills through a family-based physical activity program: results from the “Active 1 + FUN” randomized controlled trial

**DOI:** 10.1186/s12966-021-01160-5

**Published:** 2021-07-17

**Authors:** Amy S. Ha, Chris Lonsdale, David R. Lubans, Florrie F. Ng, Johan Y. Y. Ng

**Affiliations:** 1 Department of Sports Science and Physical Education, Kwok Sports Building, The Chinese University of Hong Kong, Shatin, Hong Kong; 2 Institute for Positive Psychology and Education, Faculty of Health Sciences, Australian Catholic University, 33 Berry Street, North Sydney, NSW 2060 Australia; 3 Priority Research Centre for Physical Activity and Nutrition, Faculty of Education and Arts, University of Newcastle, Callaghan, NSW 2308 Australia; 4 Department of Educational Psychology, Ho Tim Building, The Chinese University of Hong Kong, Shatin, Hong Kong; 5 Faculty of Education, The Chinese University of Hong Kong, Shatin, Hong Kong

**Keywords:** Accelerometry, Co-physical activity, Moderate-to-vigorous physical activity, Self-determination theory, Hierarchical linear models, Health-related quality of life

## Abstract

**Background:**

Physical activity is related to many positive health outcomes, yet activity levels of many children are low. Researchers have suggested that family-based interventions may improve physical activity behaviors of both children and their parents. In this study, we evaluated the “Active 1 + FUN” program, which was designed based on tenets of self-determination theory. Intervention components included free sporting equipment, ten coach-led workshops and activity sessions, and one booster session.

**Methods:**

We evaluated the intervention program using a randomized controlled trial. One hundred seventy-one families were randomly allocated to either an experimental group or a wait-list control group. Participants were exposed to program contents over a nine-month period, while families in the control did not receive any form of intervention. Measured constructs included moderate-to-vigorous physical activity, co-physical activity behaviors, fundamental movement skills, BMI, and several self-reported questionnaire outcomes. Hierarchical linear modeling was used to compare changes in measured outcomes across the two groups.

**Results:**

No significant intervention effects were found for children’s and parents’ accelerometer-measured moderate-to-vigorous physical activity, or their co-physical activity. However, in terms of children’s fundamental movement skills, a significant Time*Group interaction (*B* = 0.52, 95% CI [0.07, 0.96] for Times 1 to 2; *B* = 0.24, 95% CI [0.01, 0.48] for Times 1 to 3) in favor of the experimental group was found.

**Conclusions:**

Results suggested that the “Active 1 + FUN” program was effective in improving children’s fundamental movement skills. Additional research is needed to examine how family-based initiatives could effectively improve physical activity behaviors too.

**Trial registration:**

ANZCTR, ACTRN12618001524280. Registered 11 September 2018, https://www.anzctr.org.au/Trial/Registration/TrialReview.aspx?id=375660.

**Supplementary Information:**

The online version contains supplementary material available at 10.1186/s12966-021-01160-5.

## Background

Physical activity (PA) is associated with beneficial physical and psychological health, [1–4], and also academic performances in children [5, 6]. However, physical inactivity is prevalent worldwide and may cause severe negative health consequence [7, 8]. Increasing PA and reducing inactivity should therefore be a priority, and efforts should be directed at individuals at all ages, including childhood. To tackle this issue, researchers have adopted school-based approaches to increase children’s PA and related outcomes with some level of success [9, 10]. However, as children’s behaviors and habits are mainly shaped or affected by parents [11], family-based methods can also be effective in promoting PA for this age group [12]. Furthermore, parents can enjoy the benefits of PA as well by being active together with their children. Engaging in fun activities together may also improve parent-child relationships.

### Self-determination theory

Based on self-determination theory (SDT [13, 14]), positive behavioral outcomes and well-being of individuals are supported when individuals’ basic psychological needs for competence, autonomy, and relatedness are fulfilled. By contrast, the frustration of these needs would result in psychological ill-being [15, 16]. Competence refers to a sense of ability to perform a skill or to complete a task, while autonomy concerns a sense of volition and choice in the behavior. Relatedness refers to the perceived presence of individuals who one cares for in relation to the behavior. Researchers have shown that SDT-based interventions can promote beneficial outcomes in terms of individuals’ behavior and well-being [17, 18]. Apart from the contents, the way intervention components are delivered is also important in determining the success of a program. For instance, need supportive instructional methods are related to need satisfaction of recipients, which in turn is related to PA intention and more adaptive forms of motivation [19]. By contrast, controlling behaviors of coaches or a need thwarting environment created by instructors may have negative impact on participants’ affect and behaviors [20, 21]. Therefore, the ability to support, and avoid frustrating, participants’ basic need should be considered a critical element in intervention design.

### Fundamental movement skills

Research has shown that the need for competence could be the most salient need, relative to autonomy and relatedness, in relation to PA behaviors [22]. Although actual competencies pertaining motor skills may, or may not, directly translate to perceptions of competence [23, 24], the psychological need could be supported through proper instruction of such skills. Within the context of PA participation, fundamental movement skills (FMS) [25] are a set of skills which reflect the actual motor competence of children. By having better FMS, children might find it easier to pick-up new sport-related skills, which may enhance their perceived competence towards PA. Specifically, FMS encompass locomotor (e.g., running and jumping), ball (e.g., throwing and catching), and stability (e.g., balancing) skills [26]. Results from systematic reviews and meta-analyses has suggested that FMS may be related to PA and health-related fitness in children [27, 28]. However, in a more recent meta-analysis, researchers found that the link between FMS and PA was inconsistent in children [29]. Nevertheless, due to the potential positive impacts of developing better FMS in children, providing quality instruction in these skills was considered a potentially important intervention element in the current study.

### Current study

In this study, we examined the effectiveness of “Active 1 + FUN”, a family-based, SDT-driven PA intervention that was designed to increase moderate-to-vigorous physical activity (MVPA) and co-activity of children and their parents. The detailed rationale and procedures of the study were presented in a published protocol [30]. In “Active 1 + FUN”, we embedded SDT principles across multiple levels of intervention design. First, activity sessions of the intervention were designed to improve participant’s motor skills (competence), provide experiences and choices for fun and engagement activities (autonomy), and build relationships between family members and other participants (relatedness). Secondly, participants were also provided with key SDT concepts during intervention workshops, with tips on how they could support other family members’ basic needs in relation to PA.

A randomized controlled trial was conducted to examine the effectiveness of the “Active 1 + FUN” intervention. Participants were randomly allocated to either the experimental or wait-list control group. Intervention effectiveness was evaluated based on the outcomes of children and parents’ MVPA, and the amount of co-activity of parent-child dyads. Intervention effects on other related outcomes, including participants body mass index (BMI), FMS, need satisfaction and frustration, and well-being, were also evaluated. These constructs were chosen as they represent various constructs within the SDT process model [14]. We hypothesized that participants in the experimental group, compared to those in the control group, would demonstrate more improvements in beneficial outcomes after receiving the intervention. To examine both the short- and long-term effects of the intervention, all outcomes were measured at baseline, immediately after the intervention, and 1 year after baseline.

## Method

### Study design

A randomized controlled design with one experimental and one control group was employed to examine the effectiveness of the “Active 1 + FUN” intervention. Prior to the start of the study, parents of all participating families provided written informed consent on behalf of themselves and their children. The protocol of the trial was reviewed and approved by an ethical review committee of the lead author’s university, and prospectively registered at ANZCTR (registration number: ACTRN12618001524280).

### Sample size calculation

Based on an expected intervention effect of *d* = 0.5 on children’s MVPA [31], we calculated the required sample size using G*Power 3.1.7 with an alpha level and power of 0.05 and power of 0.8. The required sample size of the trial was calculated to be 128 families. Based on an expected accelerometer adherence rate of 70% and an estimated program dropout rate of 10%, the target recruitment sample size was calculated at 204.

### Participants

**Fig. 1:**
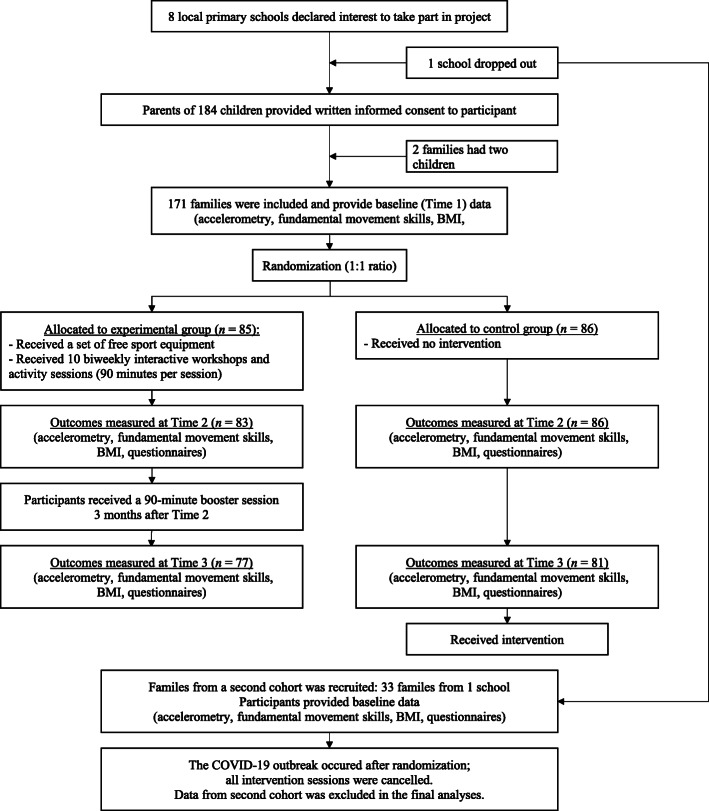
A flow diagram representing the randomized controlled trial

In September 2018, eight local primary schools in Hong Kong responded to our invitation and helped recruit families to take part in the trial. One school dropped out before baseline measures without providing a reason. A total of 171 families from seven schools were recruited and completed all data collection in the first year (from September 2018). A second cohort of 33 families from one school was recruited and began the trial in September 2019. Unfortunately, data collection and intervention delivery to the second cohort were severely affected due to the outbreak of COVID-19 between January to September 2020. As a result, data from the second cohort was not included in the final analyses. The final sample consisted of 171 families from seven schools. The number of families recruited per school ranged from 20 to 30 (mean = 24.4 families). Children were eligible to take part if they were in Grades 3 to 5. Siblings of the same family were allowed to participate together if all children were in Grades 3 to 5. Siblings in other grades were considered ineligible. Two participating families had two children meeting the criteria and took part in the study together *(*i.e.*, in the same study arm)*. Other families only had one child taking part in the study. Participating children had a mean age of 10.0 years; 69 (40%) were female. Data were collected as matched parent-child pairs. Hence data were collected from one parent per child. At Time 1, 70% of parents who provided data for analyses were mothers. A demographic breakdown of participating families is shown in Table 1. A flow diagram showing the procedures of the final included sample is shown in Fig. 1. A CONSORT checklist is also included as supplementary material.

**Table 1:** Demographic background of participating families

	Experimental group (*n* = 85)	Control group (*n* = 86)	*p*
Children age	9.93	9.98	.75
Children sex			.60
Male	49	53	
Female	36	33	
Parent age			.86
29 years or younger	1	0	
30–34 years	5	7	
35–39 years	17	18	
40–44 years	25	26	
45 years or above	15	16	
Did not report	22	19	
Parent education			.04
Primary or below	1	2	
Secondary	38	33	
Non-degree	7	19	
Degree	13	5	
Master or above	4	6	
Did not report	22	21	
Employment status			.57
Housewife	29	31	
Full-time	22	18	
Part-time	11	15	
Unemployed	0	1	
Retired	0	0	
Did not report	23	21	
Marital status			.18
Married	59	56	
Single	0	2	
Divorced	2	6	
Widowed	0	1	
Did not report	24	21	
Household monthly income			.33
HK$0–9999	4	10	
HK$10,000 – 19,999	20	13	
HK$20,000 – 29,999	13	11	
HK$30,000 – 39,999	9	7	
HK$40,000 – 59,999	11	12	
HK$60,000 – 79,999	1	6	
HK$80,000 – 99,999	2	2	
HK$100,000 or more	1	1	
Did not report	24	24	
Number of children in household			.19
1	18	21	
2	32	38	
3	13	6	
Did not report	22	21

### Procedures

Families who provided informed consent were invited to take part in data collection sessions at baseline (Time 1; September 2018), at the end of the intervention period (Time 2; approximately 6 months after Time 1), and 1 year after baseline (Time 3; September 2019). The included measures for children and parents were taken at all data collection time points. Families who attended data collection sessions received a HK$100 gift voucher (approximately US$12.80) per time point. Randomization procedures took place one to two weeks after baseline measures at the respective schools. Randomization was conducted at the family level. A family that included more than one child was considered as one unit within the randomization. Specifically, participants drew sealed envelopes to determine whether they were allocated to the experimental group or the control group. A 1:1 ratio was used for group allocation. Families, including children and their parents, randomized into the experimental group received the intervention in the same year. Families allocated to the control group only received the intervention after Time 3 measures were taken.

### Intervention

The intervention consisted of ten 30-min workshops followed by 60-min activity classes, led by two coaches in each session. Families allocated to the experimental group were invited to attend intervention sessions. One or both parents attended sessions together with the child. Due to parents’ work commitments and availability, a child may be accompanied by their father or mother, or both, during different intervention sessions. Coaches responsible for leading the sessions were trained specifically for this study. We provided 12 h of face-to-face training for all coaches over 4 months (three separate session between September and December 2018). Essentially, coaches who were responsible for intervention delivery were trained to lead intervention sessions based on the SAAFE principles [32], which were derived from tenets of SDT. The principles highlight the importance of coach-led sessions being supportive, active, autonomous, fair, and enjoyable. We also continued communication and provided feedback to coaches through mobile phone chat groups (i.e., WhatsApp).

Intervention sessions took place every 2 to 3 weeks, excluding school holidays. Therefore, the intervention period was approximately 6 months in duration. The 30-min workshops took place inside classrooms within the schools. During the workshops, participants were provided with knowledge in terms of health benefits of regular PA, parenting tips, and principles of SDT through a story-telling approach. Since these topics may involve theoretical concepts that might be complex to children and parents, we used story-telling to facilitate understanding and improve memorability of key principles [33, 34]. Apart from increasing participants’ knowledge, the workshops were designed with a goal of raising the importance of basic need satisfaction, and how parents can be more need supportive, and less controlling. To promote self-monitoring, participants also received a logbook to record any activities children and parents did together. Coaches also led participants to share interesting activities or stories they recorded on their logbooks with other attending families. Coaches also provided individual feedback to all families individually.

The activity sessions focused on the instruction of FMS, by incorporating different types of parent-and-child activities and games. These sessions took place in school halls or playgrounds to allow more open space for activities. Some activities and games were designed around a set of free equipment participants received. These included a sponge flying disc, soft volleyball, skipping rope, a pair of rackets, and some sponge balls. After the tenth session, participants were invited to attend a booster session approximately 3 months afterwards. The structure of the booster session was similar to previous sessions. That is, it included a 30-min workshop and sharing session, and the remaining 60 min were spent on group activities. During the workshop session, coaches reinforced the key messages delivered throughout the first ten sessions and invited participating families to share experiences they had after the tenth session. For the activity session, coaches invited parents and children to take more initiative in choosing what activities to do, even allowing some of them to lead group games.

### Measures

Measures for the primary and secondary outcomes of the trial were determined a priori and were presented previously [30] and registered at ANZCTR (ACTRN12618001524280). Further details of the protocols used are presented below.

#### Primary outcomes

The primary outcomes of the trial was children’s MVPA, which was measured using ActiGraph wGT3X-BT accelerometers. Participants were administered devices and were asked to fasten them to their waist on five consecutive days, except during water-based activities. Data from one day was considered valid if the device was worn for at least 8 h [35]. For the calculation of daily MVPA, only cases with two or more valid weekdays plus at least one valid weekend day were included [36, 37]. Evenson et al. [38] cut-points were used for activity intensity characterization for children data. One-second epochs were used for characterization of individual MVPA data [39].

#### Secondary outcomes

Parents’ MVPA, and co-PA behaviors of children and parents were secondary outcomes of the trial. These were also measured using ActiGraph wGT3X-BT devices. Monitors for parents were administered and returned together with children’s devices. The same criteria for determining valid wear time were used for parent MVPA data. Activity intensity classification was based on Freedson et al.’s [40] cut points. To measure participants’ co-PA, we utilized the proximity feature of the wGT3X-BT devices. This function provides information in terms of when two devices, in other words the child and parent, were in proximity (approximately within 10 m). Based on pilot information gathered from trialing intervention activities, we defined co-PA as when the following three conditions were met within a 60-s epoch on days when both the child and the parent had valid data: (i) the pair of devices had proximity signal, (ii) the child was doing MVPA, and (iii) the parent was doing light or MVPA. As co-PA measures required having valid accelerometer data for both the parent and the child on the same day, we relaxed the validity criteria for co-PA calculation to ensure there was sufficient valid data for analyses. Therefore, we considered co-PA data to be valid if both the child and parent provided sufficient data (i.e., at least 8 h) on at least 1 day.

Additional secondary outcomes included children and parents’ BMI, FMS, and self-reported questionnaire variables. Participants’ height and weight were measured using a stadiometer and electronic scale, respectively. These measurements were used to calculate the BMI of participants. We did not use adjusted BMI in this study because researchers have suggested that unadjusted BMI is a better indicator for adiposity of children in longitudinal studies [41]. Fundamental movement skills proficiency was measured using protocols from the Test of Gross Motor Development (TGMD-3 [42];). Specifically, all participants’ performances in four skills (overhead throw, forehand strike, catch, and kick) in two trials were video-recorded and rated by the same research assistant. Based on the TGMD-3 protocol, scores for catch had a maximum score of six, while the other skills had a ceiling of eight. Therefore, to calculate the overall FMS score, catch scores were scaled up by a multiplicative factor of 4/3 before conducting the analyses.

Children also completed self-report questionnaires that included measures for perceived autonomy support from parents (6 items, Cronbach alpha [α] = .79) [43], perceived control from parents (7 items, α = .71) [43], basic need satisfaction and frustration (9 items for either subscales, α’s for need satisfaction / frustration = .80 / .85) [44], and health-related quality of life (10 items, α = .72) [45]. Parents also reported their degree of autonomy support provided (6 items, α = .80) [43], controlling behaviors (7 items, α = .73) [43], need satisfaction and need frustration (α’s for need satisfaction / frustration = .79 / .84) [44], and their well-being (8 items, α = .91) [46].

### Data analyses

The interrelations between measured variables were examined using Pearson correlation. Hierarchical linear models were evaluated using MLwiN v2.26 [47]. We used two-level (time nested within family), random intercept and random slope models to evaluate the effectiveness of the intervention. One advantage of using hierarchical linear models to evaluate longitudinal data is that participants data will be retained for analyses even if they have missing data at one or more time points. By contrast, when using analysis of variance approaches, missing data would lead to a reduced sample size, and thus lessened statistical power. Specifically, we compared the differences between the experimental and control groups in terms of changes in all measured outcomes. The significance of the Time, Group (experimental versus control group), and Time*Group terms in the regression models were examined. A significant Time*Group term would imply the existence of an intervention effect. The analyses were conducted for changes from Time 1 to Time 2, and from Time 1 to Time 3, respectively.

## Results

### Preliminary results

The majority of the participating families were from low-to-mid social economic classes with married parents. The demographic backgrounds of participants in the experimental and control groups were similar (Table 1). In terms of attendance to intervention sessions, participants in the experimental group averaged an attendance rate of 79% to workshops and activity sessions. Children attended the majority of sessions with their mothers (70%), followed by father (26%), and both parents together (4%). The booster sessions were attended by 78% of the families.

The descriptive statistics of all measured outcomes, at all time-points, are shown in Table 2. Relative to families that provided data at the first time point, 90.8 and 72.3% of participants provided data at Times 2 and 3, respectively. We examined whether the baseline measures differed between participants who were, versus were not, retained at Times 2 and 3, respectively. No difference in any of the measured outcomes were found. The Pearson correlations between measured variables are presented in Table 3. As objectively measured co-PA is a relatively new measure in the research field, we explored how it was associated with other constructs. We found that co-PA was positively associated with children’s perceived autonomy support (*r* = .15), need satisfaction (*r* = .11), and negatively with BMI (*r* = −.09). We also found that it was related to parent outcomes, including autonomy support (*r* = .17), need satisfaction (*r* = .18), need frustration (negatively, *r* = −.18), and well-being (*r* = .16).

**Table 2:** Descriptive statistics of measured variables at all time points

	Time 1				Time 2				Time 3			
	Exp		Con		Exp		Con		Exp		Con	
	*n*	*M*	*n*	*M*	*n*	*M*	*n*	*M*	*n*	*M*	*n*	*M*
**Primary outcomes**												
Student MVPA (min/day)	68	47.24	68	46.77	38	62.29*	44	54.62*	25	43.69	34	45.56
Parent MVPA (min/day)	56	50.33	63	49.35	50	63.83	67	58.20	36	58.24	46	56.92
Co-physical activity (min/day)	61	9.27*	65	6.52*	50	10.39*	60	6.65*	55	4.97	68	4.76
**Secondary outcomes – Student variables**												
FMS (score out of 8) ^a b^	82	5.09	79	5.21	71	5.27*	74	4.93*	55	5.33	60	4.99
BMI (kg m^−2^)	83	16.82	77	17.20	74	17.60	74	17.39	57	17.22	61	17.71
Perceived need support	82	3.49	80	3.33	72	3.42	72	3.26	56	3.32	61	3.30
Perceived control	82	3.03	80	3.15	72	3.13	72	2.96	56	3.00	61	3.01
Need satisfaction	82	4.95	80	4.93	72	4.99	72	4.93	56	4.88	61	5.13
Need frustration ^a^	82	3.20	80	3.26	71	3.72*	72	3.18*	56	3.31	61	3.07
HRQoL ^b^	78	3.59	75	3.42	71	3.50	71	3.36	55	3.40	61	3.52
**Secondary outcomes – Parent variables**												
FMS (score out of 8)	79	5.01	81	4.91	69	4.84	71	4.49	54	5.10	58	4.83
BMI (kg m^−2^)	83	22.86	79	23.65	73	23.04	73	23.38	54	22.99	60	23.03
Provided need support	81	4.04	79	3.98	71	3.99	74	3.89	51	4.04	56	3.86
Exerted control	81	2.82	79	2.80	71	2.72	74	2.80	51	2.73	56	2.82
Need satisfaction	81	5.53*	79	5.27*	71	5.50	74	5.28	51	5.51*	56	5.18*
Need frustration	81	2.68	79	2.90	71	2.50*	74	2.99*	51	2.65*	56	3.20*
Flourishing	78	5.79	73	5.58	67	5.75	69	5.51	46	5.77	56	5.44

*Exp* Experimental group, *Con* Control group, *MVPA* Moderate-to-vigorous physical activity, *FMS* Fundamental movement skills, *BMI* Body mass index, *HRQoL* Health-related quality of life

^a^ Significant Time*Group effect from Time 1 to Time 2; ^b^ Significant Time*Group effect from Time 1 to Time 3; *Significant differences between experimental and control groups at corresponding time point

**Table 3:** Pearson correlation between all measured variables

		1	2	3	4	5	6	7	8	9	10	11	12	13	14	15	16
1.	MVPA (C)																
2.	MVPA (P)	.31**															
3.	Co-PA	.38**	22**														
4.	FMS (C)	.08	.00	-.05													
5.	BMI (C)	-.05	-.02	-.09*	.16**												
6.	PAS (C)	-.04	.06	.15**	-.02	-.03											
7.	Per Control (C)	.18**	.02	-.01	-.02	-.14**	.20**										
8.	NS (C)	-.02	.00	.11*	.01	-.11*	.60**	.22**									
9.	NF (C)	.18**	.02	-.02	.04	-.01	-.02	.43**	.05								
10.	HRQoL (C)	-.06	-.01	.04	-.05	-.02	.44**	.09	.46**	.03							
11.	FMS (P)	.06	.19**	.01	.31**	.00	-.04	-.10*	-.07	-.04	.02						
12.	BMI (P)	-.15**	-.06	-.11*	-.03	.29**	-.02	-.10*	-.08	-.02	-.07	.00					
13.	AS (P)	-.06	.00	.17**	.01	-.11*	.11*	-.05	.13**	-.10*	.12*	.03	-.07				
14.	Control (P)	.10*	-.03	-.03	-.01	.06	-.12*	.33**	-.09	.21**	-.04	.04	.08	-.02			
15.	NS (P)	.05	.05	.16**	.10*	.00	.09	.02	.16**	.00	.11*	.09	.00	.45**	.00		
16.	NF (P)	.08	-.04	-.18**	-.02	.06	-.07	.04	-.02	.15**	.01	.04	.11*	-.21**	.25**	-.34**	
17.	Well-being (P)	-.03	.02	.16**	.03	.03	.14**	.00	.15**	-.01	.13**	-.01	-.09	.44**	.02	.66**	-.38**

*Note.* (C) denotes children variables, (P) denotes parent variables. * *p* < .05, ** *p* < .01

*MVPA* Moderate-to-vigorous physical activity, *Co-PA* Co-physical activity between children and parents, *FMS* Fundamental movement skills, *BMI* Body mass index, *PAS* Perceived autonomy support from parents, *Per Control* Perceived parental control, *NS* Need satisfaction, *NF* Need frustration, *HRQoL* Health-related quality of life, *AS* Autonomy support provided, *Control* Controlling behaviors

### Intervention effects on primary outcomes

We examined the intervention effects on the primary outcomes of children’s MVPA using a random-intercept, random-slope hierarchical linear model. We found no Time*Group interaction effects for these outcomes from Times 1 to 2 (*B* = 7.20, 95% CI [− 1.87, 16.27], *p* = .12), or from Times 1 to 3 (*B* = − 1.175, 95% CI [− 5.33, 2.98], *p* = .58).

### Intervention effects on secondary outcomes

In terms of parents’ MVPA, no Time*Group interaction effects were found at either Time 2 (*B* = 4.66, 95% CI [− 5.64, 14.96], *p* = .38) or Time 3 (*B* = 0.18, 95% CI [− 6.01, 6.36], *p* = .96). With regard to co-PA between children and parents, the Time*Group interaction term was not significant from Times 1 to 2 (*B* = 0.99, 95% CI [− 3.21, 5.18], *p* = .65), or from Times 1 to 3 (*B* = − 1.27, 95% CI [− 3.02, 0.48], *p* = .15).

We found a significant Time*Group effect for children’s FMS from Times 1 to 2 (*B* = 0.52, 95% CI [0.07, 0.96], *p* = .02), and Times 1 to 3 (*B* = 0.24, 95% CI [0.01, 0.48], *p* = .05). There were no intervention effects on parents’ FMS (Times 1 to 2: *B* = 0.06, 95% CI [− 0.32, 0.45], *p* = .75; Times 1 to 3: *B* = .06, 95% CI [− 0.14, 0.26], *p* = .56). With regards to children’s need frustration towards PA, the Time*Group interaction was significant from Times 1 to 2 (*B* = 0.60, 95% CI [0.02, 1.18], *p* = .04). However, this trend was not observed for the same outcome from Times 1 to 3 (*B* = 0.14, 95% CI [− 0.17, 0.46], *p* = .37). The Time*Group interaction term was significant, suggesting the presence of an intervention effect in favor of the control group, for children’s HRQoL from Times 1 to 3 (*B* = − 0.14, 95% CI [− 0.27, − 0.02], *p* = .02). The same interaction was not found from Times 1 to 2 (*B* = − 0.04, 95% CI [− 0.05, 0.32], *p* = .77). No other significant group or interaction effects were found for other measured outcomes. We repeated all analyses conducted by adding a Gender*Time*Group term to examine if gender effects might be present. However, the interaction terms were not significant.

## Discussion

Results from the randomized controlled trial suggested that children and parents in the experimental and control groups showed similar changes in MVPA patterns from baseline to post-intervention time points. Specifically, both children’s and parents’ MVPA increased from Times 1 to 2, but they remained the same between Times 1 to 3. However, the hypothesized Time*Group interaction was not found, unlike previous family-based studies [31, 48] where intervention effects were found in parents’ MVPA. This difference may be attributed to the baseline activity levels of parents. Specifically, parents from previous studies were rather inactive at baseline, whereas parents in our study averaged approximately 50 min of MVPA per day before any intervention was applied. One possible explanation is that selection bias was present as we did not strategically recruit children or parents who were inactive. Some families might have participated because they were already active or enjoyed doing PA, which could have resulted in ceiling effects.

A secondary outcome of the trial was the amount of time parents spent doing PA with their children (i.e., co-PA). In this study, we found that co-PA was related to children’s perceived autonomy support, need satisfaction, and BMI. The Time*Group interaction effect for this outcome was non-significant. However, our results also suggested that families in the experimental group performed more PA together at Times 1, when compared to their counterparts in the control group. To our knowledge, this is the first randomized trial that has utilized the proximity features of accelerometers to measure co-activity. Previous studies have largely relied on self-report methods to measure such behaviors [49, 50], which might be prone to reporting bias. Nonetheless, few studies to date have applied the proximity function in accelerometers for the measurement of co-PA [51, 52]. There is therefore no consensus on how these behaviors should be operationally defined. Nonetheless, co-activity and play between children and parents may have other social and psychological benefits [53], and we have demonstrated that the proximity feature of research accelerometers can be utilized as an objective and valid measure for co-PA outcomes in future studies.

Despite lacking evidence of intervention effects on MVPA and co-PA, a significant Time*Group effect in favor of the experimental group was found for children’s FMS. Proficiency in FMS is important as it is positively related to children’s PA engagement and cardio-vascular fitness, and negatively associated with weight status [28]. Our results suggest that the intervention can improve children’s FMS, which may have long-term benefits in PA and health [54]. Although there was a positive intervention effect, we found that the overall FMS proficiency of children were in fact decreasing from Times 1 to 3. In fact, research conducted elsewhere has also shown that children’s FMS performance may drop after reaching a certain age range [55]. Nonetheless, this finding is worrying because children in this trial have been attending normal schooling, where they also received formal physical education. This suggests that in-service physical education teachers may require additional support through professional development training in terms of pedagogical and assessment skills, knowledge in FMS, or curriculum support to adequately support students’ FMS development [56]. In particular, the physical education curriculum in Hong Kong for children of this age has just transited from one which solely focuses on FMS instruction (Grades 1 to 3) to the next phase (Grades 4 to 6) which incorporates sport-specific skills and activities [57]. Teaching of FMS per se did not form part of the curriculum. Additional research may be needed to examine whether the shift in teaching focus may have led to drops in children’s FMS competence. Yet in this study, parents, but not just children, received knowledge and instructions on FMS. This empowers parents to assess and monitor their children’s development on their children’s motor skills. Nonetheless, there is insufficient evidence from the current study to suggest whether such changes have contributed to the positive intervention effects found. Researchers may examine how involvement of parents might contribute children’s motor development in future studies.

We found a significant detrimental intervention effect (*B* = 0.60, *p* = .04) on children’s need frustration from Times 1 to 2. Previous research has shown that controlling behaviors from significant others may lead to higher levels of need frustration [58]. Nonetheless, we did not observe increased levels of parental control in the current study. Therefore, the increased levels of need frustration may be related to sources other than parents’ behaviors. For example, participating in activity classes with other children may have evoked social comparison with others, which may have highlighted children’s own shortcomings, and resulted in a reduced sense of competence. Alternatively, children who did not enjoy the intervention might have felt pressured to attend these sessions, which could also have frustrated their need for autonomy.

The current study has several strengths. First, we applied a novel approach for the measurement of parent-child co-activity. This approach could reduce potential self-report biases from questionnaire-based measures of the same construct. Second, previous interventions designed based on SDT have relied on an external agents to provide need support to participants [59]. In our study, coaches were trained to apply the SAAFE principles [32] in their instruction to support participants’ needs. By providing coaches with knowledge regarding the theoretical basis of the approach and the intervention (i.e., SDT), coaches may be more likely to endorse and apply these instruction principles. We have also closely monitored how sessions were conducted and maintained close communication with coaches to ensure intervention sessions are conducted with sufficient fidelity. For example, intervention sessions held at each site were reviewed by the authors at least once on-site, and one other time using recorded videos. Specific feedback was then shared between all coaches. In addition, we also provided participants with knowledge and key concepts of the theory using a story-telling approach, and provided tips on how these can be translated to actual practices. This approach was found to be appropriate and effective by parents [60]. One limitation of the current study was that the required sample size was not achieved due to the COVID-19 outbreak. This resulted in 15% reduction in the effective sample size. It is unclear whether some null findings of the study were related to the lack of power. However, we still found intervention effects on children’s FMS, which is an important precursor to children’s lifelong PA engagement. Participants of the study, in particular the parents, were relatively fit and motivated at baseline. This may have resulted in ceiling effects and therefore the lack of findings in terms of PA outcomes. Future research may benefit from specifically targeting parents and children who are inactive or are at risk of health issues. Finally, a one-day valid wear-time criterion was used for co-PA measures to retain a meaningful sample size for data analyses. This may not be desirable as data from a single day may not be representative. One potential solution is to increase the number of days parents and children would be asked to wear the devices. However, in our study, batteries of most deployed accelerometers would be depleted after approximately 5 to 6 days when the proximity feature was enabled. Thus, other instructions or data handling approaches may need to be developed for a more agreeable approach to collect co-PA data using accelerometer proximity.

In conclusion, results from this randomized controlled trial has suggested the “Active 1 + FUN” intervention to be effective in improving children’s FMS, but not MVPA. Preliminary evidence also suggested that parents’ and children’s co-PA behaviors may be related to positive outcomes. The modest changes found in children’s FMS are promising, yet more research is needed to examine how family-focused interventions could produce changes in children and parents’ PA behaviors [61].

## Supplementary Information

**Additional file 1.** CONSORT 2010 checklist.

**Additional file 2. **TIDieR checklist.

## Data Availability

The datasets used and/or analysed during the current study are available from the corresponding author on reasonable request.
