# Supplementary material for: Improving children’s fundamental movement skills through a family-based physical activity program: results from the “Active 1 + FUN” randomized controlled trial
Source: Int J Behav Nutr Phys Act. 2021 Jul 17;18:99. doi: 10.1186/s12966-021-01160-5 (PMC8285675; doi:10.1186/s12966-021-01160-5)
Supplement: Supplementary file 2 — Additional file 2. TIDieR checklist. [file 12966_2021_1160_MOESM2_ESM.docx]

**Recruitment procedures**

1. School recruitment
   Invitation letters were sent out to randomly selected local primary schools in batches of 100. A total of 8 schools responded positively after the third batch of letters were sent. Since there is a total of 483 primary schools in Hong Kong, the invitation letter reached approximately 62% of all eligible schools.
2. Family recruitment
   Invitation letters were sent to all eligible families in the schools who agreed to participate. Based on our inclusion criteria, students from two grades within the schools received the invitation letter. Specifically, the letter invited families to take part in a “family physical activity research study”.
   An on-site briefing session was arranged at each school (except the school that dropped out) before families confirmed their participation by providing written informed consent. The goal of these briefing sessions was to explain the design of the study and that they will be randomly allocated into either the experimental group or control group.

**Sample representativeness**

We have made attempts to minimize potential selection biases during the recruitment process. We feel that the final sample is a fair representation of families who have some interest in engaging in family exercises.

**Missing data handling**

All missing data were treated as missing in our hierarchical linear models, which does not require all participants providing scores at each time point. No imputation or other methods were used to replace missing data.
